# In vivo remineralization of dentin using an agarose hydrogel biomimetic mineralization system

**DOI:** 10.1038/srep41955

**Published:** 2017-02-07

**Authors:** Min Han, Quan-Li Li, Ying Cao, Hui Fang, Rong Xia, Zhi-Hong Zhang

**Affiliations:** 1 College & Hospital of Stomatology, Anhui Medical University, Key Lab of Oral Diseases Research of Anhui Province, Hefei, 230032, China; 2 Department of Stomatology, the Second Hospital affiliated to Anhui Medical University, Hefei, 230601, China; 3 Department of Stomatology, the Hospital of Anhui Province, Hefei, 230001, China

## Abstract

A novel agarose hydrogel biomimetic mineralization system loaded with calcium and phosphate was used to remineralize dentin and induce the oriented densely parallel packed HA layer on defective dentin surface *in vivo* in a rabbit model. Firstly, the enamel of the labial surface of rabbits’ incisor was removed and the dentin was exposed to oral environment. Secondly, the hydrogel biomimetic mineralization system was applied to the exposed dentin surface by using a custom tray. Finally, the teeth were extracted and evaluated by scanning electron microscopy, X-ray diffraction, and nanoindentation test after a certain time of mineralization intervals. The regenerated tissue on the dentin surface was composed of highly organised HA crystals. Densely packed along the c axis, these newly precipitated HA crystals were perpendicular to the underlying dental surface with a tight bond. The demineralized dentin was remineralized and dentinal tubules were occluded by the grown HA crystals. The nanohardness and elastic modulus of the regenerated tissue were similar to natural dentin. The results indicated a potential clinical use for repairing dentin-exposed related diseases, such as erosion, wear, and dentin hypersensitivity.

Human tooth is composed of the outermost enamel layer and the inner dentin−pulp complex. Dental enamel, as the hardest mineralized tissue present in the human body, approximately 96% of which is composed of well-organized crystallites of inorganic mineral nanorod-like hydroxyapatites (HAs)^1^,^2^. HA crystals, bundled in parallel with one another along the c-axis, assemble into prism structures which build up the enamel micro-structure. Dentin, the main component of teeth, located in the inner layer of the enamel and cementum, is a mineralized collagenous tissue^3^ which is composed of about 70% of mineral nanorod-like HAs, 20% of organic matter and 10% of water. The main organic compound of dentin is type I collagen, which self-assembles into fibrils to form collagen matrix scaffolds. So, the dentin has greater elasticity and lower average microhardness than the enamel. Furthermore, dentin is a porous structure with the special structure- dentinal tubules.

Dental hard tissue, both the enamel and the underlying dentin, cannot self-heal if damaged. Tooth defects are usually filled with synthetic materials, such as amalgam, ceramics, or polymer composites in clinical dentistry. These materials are not very satisfactory and can result in problems, such as trauma to sound dental tissue, dentin hypersensitivity, and interface microleakage between filling materials and dental tissue. The WHO World Oral Health Report 2003 identified restoration of tooth defects with artificial materials as a continuing global problem and serious resource burden^4^.

Recently, various cell-free biomimetic mineralization strategies have been reported, such as a hydrothermal method^5^, acidic calcium phosphate paste containing hydrogen peroxide and phosphoric acid^6^, bioactive glass^7^, surfactants^8^, nano-HA and proline^9^, amelogenin^10^, gelatin^11^, dendrimer^12^, ethylenediaminetetraacetic acid^13^,^14^, polyethylene oxide and polyacrylamide^15^, and polydopamine^16^. These cell-free biomimetic mineralization strategies have been confirmed to be capable of regenerating enamel-like or dentin-like tissue microstructures *in vitro*^17^,^18^,^19^,^20^. These methods have provided the potential for tooth defect repair via a self-healing mechanism, which is a much more ideal for use in dentistry clinics than the current traditional treatment measures. Though these methods may not be able to replace the traditional synthetic materials in treating large tooth defects, it may be potential to be used to treat micro tooth defect (such as the early caries, white spot lesions, dentin hypersensitivity) at the current stage.

There are some differences between the remineralization of enamel and dentin. The aim of remineralization of enamel lesion is to make the previous demineralized tissue remineralized (namely, put mineral back onto a lesion surface, ideally penetrating to the subsurface areas of the lesion), to ideally achieve an area more resistant to acid attack than the original enamel tissue^21^. The aim of the remineralization of dentin is to regenerate a dentin microstructure of remineralized collagen matrix, especially forming intrafibrillar hydroxyapatite, and forming hydroxyapatite to occlude the open dentinal tubules. It is much more desirable to form precipitation of minerals connecting and covering dentin surface similar to native enamel to protect dentin-pulp complex^22^. Remineralization or regeneration of dentin-like tissue is much more difficult than remineralization or regeneration of enamel-like tissue in clinics and laboratory experiment^23^.

The other thing must be noted is that, all above mentioned studies were taken out *in vitro*, wherein the biomimetic remineralization process occurred on the surface of a clean, polishing, and acid-etched tooth piece. The surroundings of the biomimetic mineralization system are quite different from the complex oral environment. In oral environment, there is a strong possibility that the biomimetic mineralization process may be interfered by the temperature, dental biofilm, salivary proteins, food debris, and bacterial products. However, few studies reported the regeneration process in the human oral environment except for one study^24^, in which the effectiveness of a biomimetic mineralization system was evaluated in patients with dentin sensitivity. However, their assessment methods were subjective, and the structure and composites of the generated substance was not characterized, so that it cannot prove the regeneration of tooth-like tissue. Therefore, the validity of the cell-free biomimetic mineralization strategies in clinical application need further evaluate.

In our previous studies, an agarose hydrogel biomimetic mineralization system was developed for regenerating dental hard tissue *in vitro*^21^,^22^,^23^,^25^. It is reported that this mineralizing system was able to duplicate the dentin microstructure, to occlude the open dentinal tubules, and to regenerate enamel-like tissue covering the remineralized dentin surface. The aim of the present study was to investigate the effectiveness of the agarose hydrogel biomimetic mineralization system in a dentin exposed animal model for its potential clinical translation. We hypothesize that this system has an ability to induce dentin collagen matrix calcifying, occlude the open dentinal tubules, and regenerate enamel-like tissue covering the remineralized dentin surface *in vivo*.

## Results

### Assessment of the structure of regenerated tissue by X-ray diffraction (XRD)

**Figure 1:**
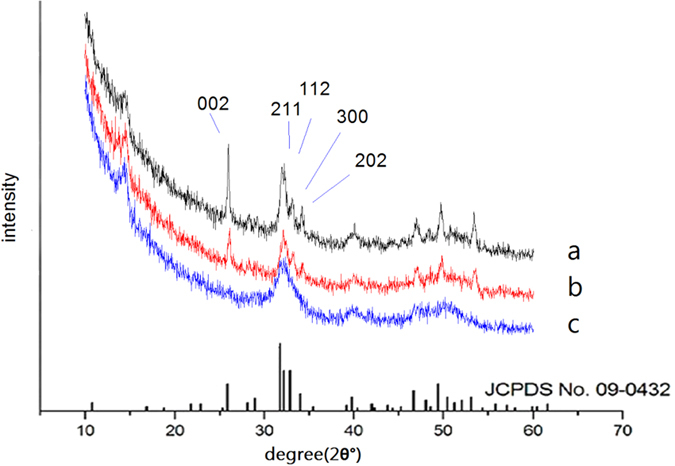
XRD spectra of the precipitates formed on dentin after three (**a**) and one (**b**) cycles of mineralization, and natural tooth (**c**). The lower spectra are the standard peaks for hydroxyapatite (HA) (JCPDS no. 09–0432).

The typical film-XRD patterns of the natural dentin, one, and three cycles of mineralization dentin are shown in Fig. 1. The main detected diffraction peaks (002) at 2θ = 25.9°, (211) at 2θ = 31.9°, (112) at 2θ = 32.2°, (300) at 2θ = 33.0°, and (202) at 2θ = 34.1° matched well with those standard peaks for hydroxyapatite (HA) (JCPDS no. 09–0432), suggesting that the precipitates generated on the surface of dentin were HA crystal.

The 002 (c-axis) peak (at 2θ = 25.9°) was stronger than the peaks of 112, 211, and 300 (a-axis) in the sample of three cycles of mineralization, indicating that the HA crystals were oriented along their c axis and well crystallised. The results are consistent with the following scanning electron microscopy (SEM) analysis.

Compared with the broad diffraction peaks of the natural dentin, the diffraction peaks around 2θ = 31.9°–33.0° were split and clear, indicating that the regenerated HA crystals exhibited better crystallinity than the natural dentin.

### Assessment of the morphology of regenerated tissue by SEM

**Figure 2:**
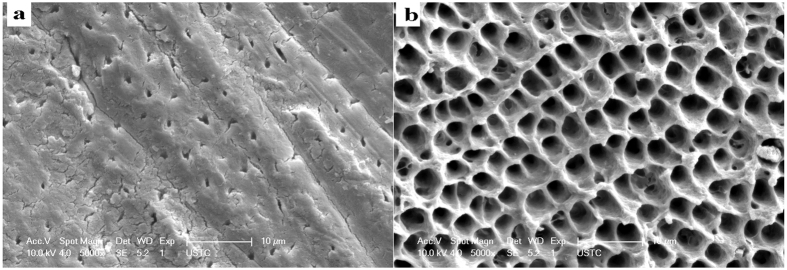
SEM micrographs of the surface of nature dentin showing smear layer covering the dentinal tubules (**a**) and the dentin surface after 37% H_3_PO_4_ etching for 20 s showing the smear layer was removed and the dentinal tubules were enlarged (**b**).

The surface of exposed dentin before acid etching showed typical dental tubules and intertubular dentin structure with some smear layer adsorbed on the surface and occluding the dentinal tubules (Fig. 2a). After 37% H_3_PO_4_ etching for 20 s, the surface of dentin was demineralized with exposed collagen matrix and enlarged dental tubules (Fig. 2b).

**Figure 3:**
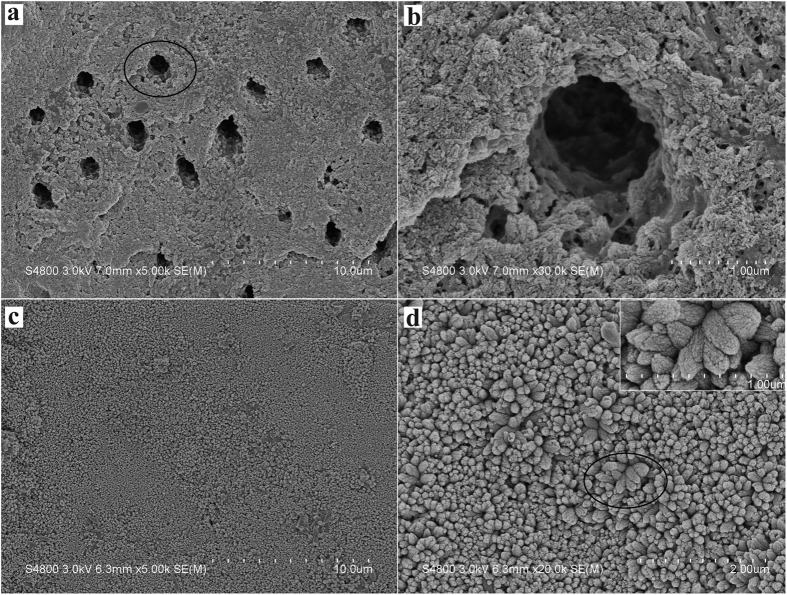
SEM micrographs of the precipitates formed on dentin after one (**a** and **b**) and three (**c** and **d**) cycles of mineralization viewing from the surface. (**b** and **d**) Are the magnification of (**a** and **c**), respectively. Inset is the magnification of subpanel (**d**).

For the experimental sample of mineralization, after 1 cycle of mineralizing, the surface of acid-etching dentin was almost covered by a layer of HA crystal particles. HA particles deposited within the collagen matrix and onto the surface of collagen matrix, and combined well with the collagen fibers. The well of dentinal tubules was also deposited by HA particles, and some dentinal tubules were partly occluded (Fig. 3a,b). After 3 cycles of mineralizing, the surface of the dentin was fully covered by HA crystals. Dentinal tubule structure was occluded and could not be seen. The oriented crystals evenly parallel grew on the dentin surface with a crystallographic c-axis perpendicular to the dentin surface, and tightly packed together to form a dense, uniform HA crystals layer (Fig. 3c,d), which was similar to our previous finding^23^,^25^. Although it was not exactly the same as the natural enamel crystals in structure, to some extent, the regenerated tissue was quite similar to the enamel when considering the similar composition and structure to the enamel and the similar protective functions to the dentin.

**Figure 4:**
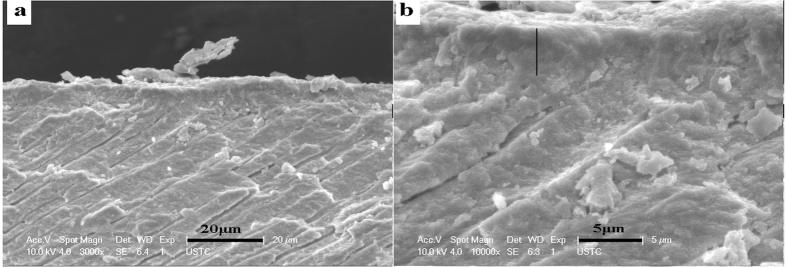
SEM micrographs showing the binding between the remineralization dentin and regenerated tissue after three cycles of mineralization viewing from the transverse section. (**b**) Is the magnification of (**a**).

Viewing from the transverse section, the thickness of the oriented densely parallel packed HA layer was approximately 4 μm after 3 cycles of mineralization (Fig. 4). The interface between the regenerated tissue and the underlying dentin showed a tight fusion (Fig. 4b). The precipitates on the dentin surface and the substrate of remineralized dentin were tightly integrated, such that the interface was hardly distinguishable. Thus, we confirmed that the binding between the precipitated HA crystals and the dentin tissue was reliable.

**Figure 5:**
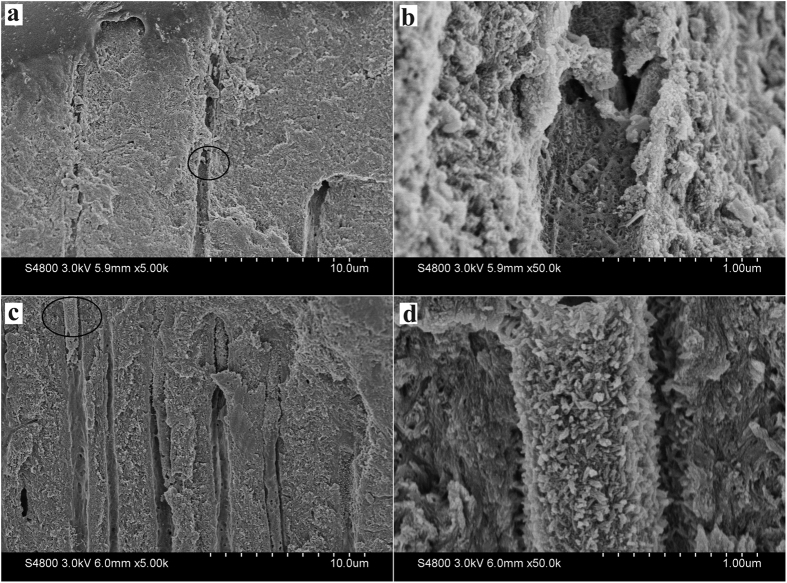
SEM micrographs of the precipitates formed on dentin after one (**a** and **b**) and three (**c** and **d**) cycles of mineralization viewing from the transverse section. (**b** and **d**) Are the magnification of (**a** and **c**), respectively.

Part of dentinal tubules were partly occluded by the grown HA crystals after one cycle of mineralization (Fig. 5a,b), whereas almost dentinal tubules were fully occluded by the enamel prism-like grown HA crystals after three cycles of mineralization (Fig. 5c,d).

**Figure 6:**
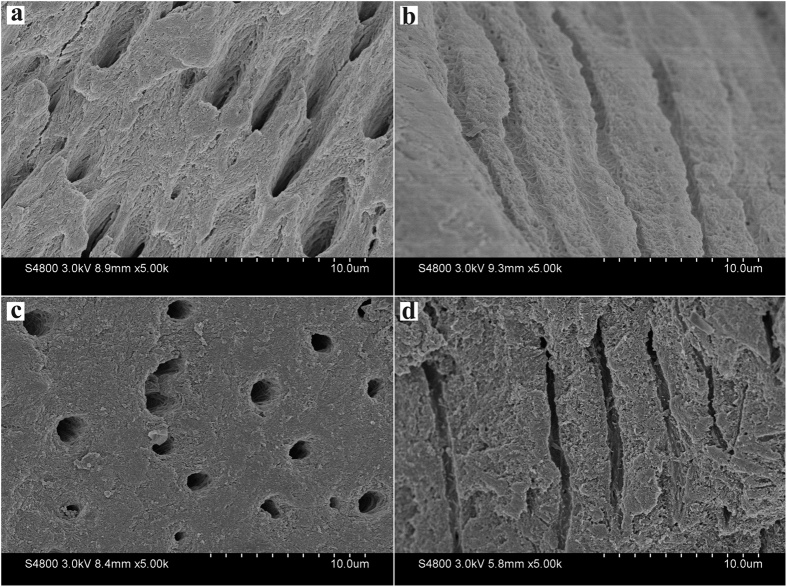
SEM micrographs of the control samples without mineralizing system after one (**a** and **b**) and three (**c** and **d**) cycles of mineralization. (**a** and **c**) Viewing from the surface; (**b** and **d**) viewing from the transverse section.

For the control samples without mineralizing system, little mineralization happened and only some film-like matrix was found on the dentin surface at the same time of one or three cycles of mineralization (Fig. 6).

### Assessment of the mechanical property of regenerated enamel by nanoindentation

The elastic modulus and nanohardness of natural dentin, acid-etched dentin, and the regenerated tissue after three cycles of mineralization are shown in Table 1. The elastic modulus and nanohardness of the regenerated tissue were similar to the untreated natural dentin. No statistically significant differences were observed in the elastic modulus or nanohardness between the regenerated tissue and the untreated dentin group (p > 0.05). Meanwhile, the etched dentin showed a significant decrease in elastic modulus and the nanohardness compared with the untreated natural dentin (p < 0.05).

## Discussion

**Figure 7:**
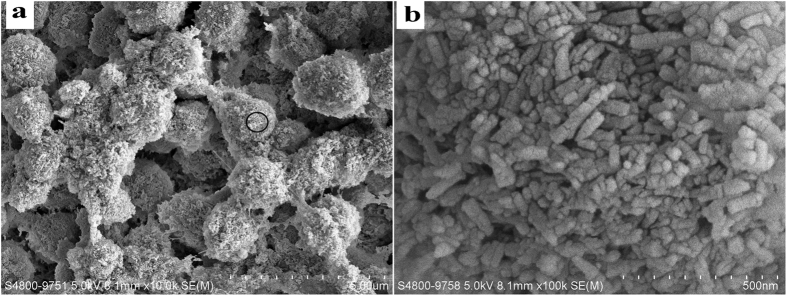
SEM micrographs of the agarose fiber-nanoscale-amorphous calcium phosphate complex precursors (**a** and **b**). Subpanel (**b**) is the magnification of subpanel (**a**).

**Figure 8:**
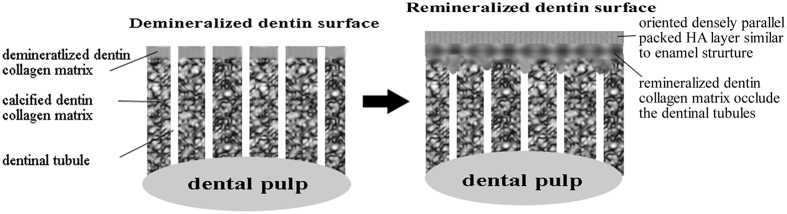
A schematic diagram of the process of the mineralization.

Human enamel and dentin were formed under the control of organic matrix environment^19^. Inspired by the molecular mechanism of organic-matrix mediated biomineralization, the regeneration of dental tissue *in situ* with a biomimetic cell-free stratagem has been recently proposed. The organic matrix acts as template in a biomineralization process and controls the mineral crystallites through the molecular interaction between the polymer and minerals with a sequestering mechanism. The ion or cluster binding in the biomimetic mineralization system assembles into amorphous primary particles at the organic surface, and then the amorphous primary particles build up and form the oriented crystallization^26^. The agarose hydrogel biomimetic mineralization system used in the present study mimicking the gel-like organic matrix environment of natural tooth formation has successfully induced enamel-like tissue regeneration on the surface of the enamel and dentin under laboratory conditions^21^,^23^. Agarose is a natural polysaccharide and consists of a linear polymer with repeating units of d-galactose and 3,6-anhydro l-galactose. In the agarose hydrogel system, agarose loaded with calcium and phosphate ions acted as an organic matrix template for biomimetic mineralization to form the agarose fiber-nanoscale-amorphous calcium phosphate complex precursors^27^. This phenomenon was detected (Fig. 7) in the study, and had been proved by our previous study^21^,^23^. Meanwhile, the agarose hydrogen acted as a reservoir to replenish mineral precursors, and the restricted space in the gel network may confer a uniform and controllable size of the fiber-nanoscale-amorphous calcium phosphate complex. The amorphous primary particles^28^,^29^,^30^, aggregated and assembled, resulting in the demineralized collagen fibrils calcified and the dentinal tubules occluded. After HA crystals subsequently nucleated and gradually grew on the calcified collagen fibrils, with their c-axis aligned parallel to each other and perpendicular to the surface of the dentin, an oriented densely parallel packed HA layer was formed with a similar structure to enamel^23^. The mineralization process was diagrammed in Fig. 8.

The biomimetic mineralization was conducted *in vitro* on clean, sterile, and pollution-free dental slices wherein the mineralization microenvironment was stable and the process of the mineralization was conducted without any disturbance. However, when we try to push the biomimetic mineralization system into clinical use, some important issues should be taken into consideration.

The first issue was on how to hold the hydrogel on the teeth surface stably and isolate it from the interference of oral environment. We designed a custom tray to load the hydrogel and kept the hydrogel stable in the mouth. The custom tray can be made easily by vacuum formed retainer appliance in clinical application. The second issue was on how to avoid the interference of oral biofilm formed on tooth surface after daily activities, such as eating and drinking. Although this concern is a key issue for the biomimetic mineralization method in clinical application, related reports are lacking. After a cycle, the rabbits were fed with food and water, and the dental surfaces coated with deposits were easily stained by dental bacterial plague, soft dirt, and food residue after these daily activities. Therefore, the stained dental surface layer must be managed properly before refreshing the hydrogels and starting a new mineralization, to keep the enamel-like tissue regenerated on the former deposition but not on the organic dental biofilm or debris. During our experiment, we brushed the surface with soft toothbrush and toothpaste for 3 min and rinsed with 3% sodium hypochlorite^31^,^32^ and large quantities of deionized water to remove the smear layer before refreshing the hydrogels. This method can be performed in clinic and home care once this system is used in clinics. Of cause, some other simple methods can be exploited in the future. The third issue was on if the mechanical friction, such as chewing and brushing, would seriously destroy the regenerated mineralization tissue. The result demonstrated that mechanical friction did not have a critical impact on the regenerated mineralization tissue. The conjunction condition of the interface demonstrated the excellent binding between the regenerated tissue and dentin substrate (Fig. 4). The fourth issue was on how long to keep the device in contact with the tooth. In laboratory study, mineral ions are fed constantly for nearly 24 h daily (one cycle), with almost no intervals. However, it is unrealistic to wear a tray filled up with agarose hydrogel for 24 h every day in clinical application. Herein, the application of mineralization was conducted and lasted 8 h daily (a cycle) in rabbit’s mouth. The wearing time of 8 h was intended to correspond with human’s sleep time. Thus, people could wear the application of mineralization in their mouth overnight in clinical use.

From above discussion, we may confirm the validity of this new technique in clinical application.

## Materials and Methods

### Preparation of the agarose hydrogel biomimetic mineralization system

The agarose hydrogel biomimetic mineralization system was presented in our previous study^23^.

Calcium chloride (CaCl_2_) agarose gel was prepared by dissolving agarose powder (BioWest regular agarose G-10, Gene Company, Origin, Spain) (1.0 g) into 100 mL of 0.13 M CaCl_2_ solution. The 0.13 M calcium chloride solution (pH adjusted to 6.5 using 0.1 M HCl and 0.1 M NaOH) was prepared by dissolving CaCl_2_·2H_2_O in deionized water.

Na_2_HPO_4_ hydrogel containing 500 ppm fluoride was prepared by mixing agarose powder (1.0 g) into 100 mL of 0.26 M Na_2_HPO_4_ (Sigma-Aldrich, St. Louis, MO, USA) solution containing 500 ppm fluoride (Sigma-Aldrich, St. Louis, MO, USA). The phosphate solution containing fluoride (pH adjusted to 6.5) was prepared by dissolving Na_2_HPO_4_·12H_2_O and NaF in deionized water to obtain a final concentration of 0.26 M Na_2_HPO_4_ solution containing 500 ppm fluoride.

Both the two mixtures were kept swelling at 25 °C for 30 min. After heating at 100 °C until completely dissolved, the two hydrogels were maintained at 50 °C before use.

### Preparation of the exposed and demineralized dentin surface on rabbit’s incisors

#### Ethics statement

This animal experiment was approved by the AnHui Medical University Experimental Animal Ethics Committee and was carried out in accordance with the National Institutes of Health guide for the care and use of Laboratory animals (NIH Publications No. 8023, revised 1978).

A total of 11 healthy, male New Zealand White rabbits aged 3 months and weighing 2.1–2.5 kg (average 2.25 kg) were included in this study. All animals were bought from the Department of Animal Experimental Central of Anhui Medical University (certificate number SYXK (Wan) 2013—004). Housed in a suitable temperature, humidity, light-controlled house, animals were carefully fed in individual coops and provided with solid diet and water ad libitum.

All rabbits were trussed up and anesthetised with an intraperitoneal injection of 10% chloral hydrate (Dopalen, Ceva Sante Animale, Libourne, France), 0.4 mg/kg^33^. The labial surfaces of maxillary and mandibular incisors were prepared to expose the dentin by removing the enamel using the dental high speed turbine handpieces with the water-cooled dental drillers and polished. Then, the exposed dentin surfaces were etched with 37% phosphoric acid gel for 20 s (Gluma Etch 35 Gel, Heraeus Kulzer GmbH, Germany), then rinsed with deionized water thoroughly. The prepared teeth were used for the following study. Maxillary incisors were used as the experimental groups for biomimetic remineralization, and the mandibular incisors were used as self-control without the biomimetic remineralization.

### Assembling the agarose hydrogel biomimetic mineralization system on the exposed and demineralized dentin surface of the rabbit model

**Figure 9:**
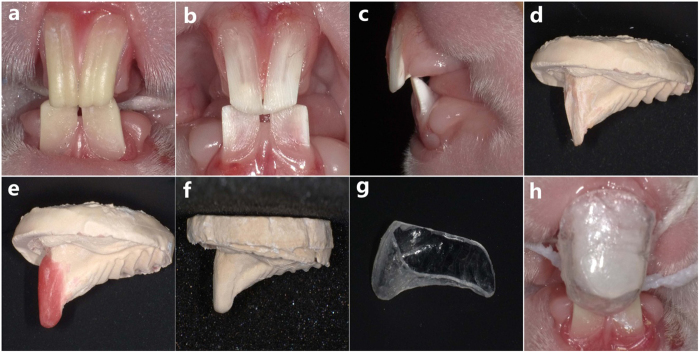
Assembling the agarose hydrogel biomimetic mineralization system on the demineralized rabbit dentin surface. (**a**) Shows the natural incisors of the rabbit. (**b** and **c**) Are the prepared incisors with dentin exposed to oral environment. (**d**) Is the plaster working model of the prepared incisors. (**e**) Shows the working teeth, with 4 mm thick wax laid on the labial surface. (**f**) Is the plaster working model of (**e**). (**g**) Shows a custom, transparent tray. (**h**) Shows the assembling of the agarose hydrogel biomimetic mineralization system.

Transparent polymer custom trays^15^ were made to load above agarose hydrogel on the labial surface of maxillary incisor. First, the alginate impressions were taken from the rabbits’ front teeth (Fig. 9a,b). Then, plaster working models were primed and shaped (Fig. 9c,d). Wax was laid on the labial surface of the maxillary incisors to maintain a 4 mm thick space for the hydrogel in the next steps (Fig. 9e). After that, a new alginate impression and plaster model were made again (Fig. 9f). Lastly, on the basis of the new plaster model, the custom tray was fabricated with the polymer transparent vacuum suction membranes (1.2 mm, Manina Vallen-Slijpen Van Coehoomstraat 20, 5916 PH Vento, Netherlands Mfg.) under the vacuum formed retainer appliance (TWDE-009, Zheng Jiang, China) (Fig. 9g).

Following these pretreatments, a 2 mm thick layer of CaCl_2_ hydrogel film was first placed onto the labial surface of maxillary incisors, and kept to gelification. Then, a tray filled up with 2 mm thick layer of Na_2_HPO_4_ hydrogel was positioned ensuring that the hydrogel covered onto the CaCl_2_ hydrogel and combined well (Fig. 9h). The application of mineralization kept stable and lasted for 8 h daily (defined as a cycle). The mandibular incisors were used as the control group, and were exposed to oral environment directly without any CaCl_2_ gel or Na_2_HPO_4_ hydrogel on the tooth surface.

After a cycle, the tray and hydrogel were removed and the rabbits were carefully fed in individual cages and then the hydrogel systems were refreshed the next day. Before refreshing the hydrogels, the teeth were brushed with toothpaste without fluoride for 3 min, rinsed with 3% sodium hypochlorite (Sigma-Aldrich, St. Louis, MO, USA) and deionized water to remove the smear layer from the exposed dentin. In the control group, the same procedure was provided to remove the smear layer from the exposed dentin in the mandibular incisor. After the one and three cycles, the rabbits were killed, and the front teeth of the experimental and control groups were extracted. Then, the pulps and periodontal tissues were removed. The labial surfaces were cut into a rectangle thin slice, sterilized, and stored in deionized water at 4 °C for the following characterization.

### Characterizing the precipitates of the biomimetic mineralization

The structure of the precipitates was characterised by X-ray diffraction (XRD) (X’Pert Pro, Philips Almelo, Netherlands).

The morphology of the precipitates on the dentin slices was evaluated by field-emission scanning electron microscopy (SEM) (Hitachi S4800, Hitachi Ltd., Tokyo, Japan; FEI, Sirion 200, USA). Before SEM analysis, the dentin slices were dehydrated with gradual ethanol, dried in the critical evaporator, and sputtered coated with gold.

The mechanical property was evaluated with a nanoindentation technique (G200, Agilent Technologies, CA, USA). Ten test points were performed on each specimen surface in each group. The elastic modulus and nanohardness were recorded. The untreated natural dentin and the acid-etched dentin were used as control. For the test, the Berkovich tip was used and calibrated with a fused-silica sample prior to evaluation. The nano-indentation test consisted of three segments: the loading segment, the peak load holding segment, and the unloading segment. The times for both loading and unloading were 15–26 s. The holding time was 10–16 s. The maximum force applied during loading and unloading was 10 gf (0.098 N). The applied load forces and the depth of penetration into the samples during the indentation were continuously monitored by a computer. The data were recorded and processed by Testworks 4 software (MTS Systems Corporation, Eden Prairie, MN, USA). The differences in the elastic modulus and nanohardness among the three groups were assessed with a one -way ANOVA, and a 5% significance cut-off level was used for the statistical analysis.

## Conclusion

This study reported the use of an agarose hydrogel biomimetic mineralization system loaded with calcium and phosphate to induce dentin remineralization and formation of a oriented densely parallel packed HA layer on dentin surface in a rabbit model *in vivo*. The results indicated a potential clinical use for repairing dentin-related diseases, such as erosion, wear, and dentin hypersensitivity.

## Additional Information

**How to cite this article**: Han, M. *et al*. In vivo remineralization of dentin using an agarose hydrogel biomimetic mineralization system. *Sci. Rep.* **7**, 41955; doi: 10.1038/srep41955 (2017).

**Publisher's note:** Springer Nature remains neutral with regard to jurisdictional claims in published maps and institutional affiliations.

## Figures and Tables

**Table 1:** Elastic modulus and nanohardness of different samples in evaluation of mechanical properties.

Sample	Avg Modulus [500–1000 nm] GPa	Avg Hardness [500–1000 nm]GPa
Dentin coated with regenerated tissue after 3 cycles	19.5 ± 3.1	1.12 ± 0.09
Untreated natural dentin	24.1 ± 1.6	0.72 ± 0.1
Etched dentin	5.5 ± 2.7	0.22 ± 0.06
